# Rabies Elimination: Is It Feasible without Considering Wildlife?

**DOI:** 10.1155/2022/5942693

**Published:** 2022-06-26

**Authors:** Krishna Prasad Acharya, Rakesh Chand, Falk Huettmann, Tirth Raj Ghimire

**Affiliations:** ^1^Animal Quarantine Office-Kathmandu, Department of Livestock Services (DLS), Budhanilkantha, Kathmandu, Nepal; ^2^Department of Veterinary Medicine, University of Cambridge, Madingley Road, Cambridge CB3 0ES, UK; ^3^-EWHALE Lab-Institute of Arctic Biology, Department of Biology & Wildlife, University of Alaska Fairbanks (UAF), AK, USA; ^4^Department of Zoology, Tri-Chandra Multiple Campus, Tribhuvan University, Kathmandu, Nepal

## Abstract

Rabies is a vaccine-preventable fatal viral disease that is zoonotic in nature. In this article, we provide a justification why the agreement of the World Health Organization (WHO), the Food and Agriculture Organization (FAO), the World Organization for Animal Health (OIE), and Global Alliance for Rabies Control (GARC) on The Global Strategic Plan to End Human Deaths from Dog-mediated Rabies by 2030 should also include a more holistic approach and ecologic views.

## 1. Introduction

Rabies is a viral (Rhabdoviridae: *Lyssavirus*), vaccine-preventable, and zoonotic disease that is 100% fatal once its clinical symptom begins. It is distributed in over 150 countries and territories with an estimated 60,000 global rabies cases per annum [1, 2]. Because rabies is an important public health issue, the World Health Organization (WHO), the Food and Agriculture Organization (FAO), the World Organization for Animal Health (OIE), and the Global Alliance for Rabies Control (GARC), all agreed on The Global Strategic Plan to End Human Deaths from Dog-mediated Rabies by 2030 [3]. It has been estimated that 99% of the human rabies cases are dog-mediated and half of them are related to those in children under 15 [4]. While the canine rabies makes up the majority of the impacts, the role of wildlife remains widely unknown in terms of a potential constant reservoir, as a source of reintroduction or spillover, thus it remains neglected in most of the rabies control programs. The aim of this comment here is to critically analyze whether we would be able to control rabies in humans and dogs without considering the key wildlife fauna around the globe.

## 2. Significance of Wildlife in Rabies Transmission

The rabies virus, a euryxenous neurotropic microbe, can infect a wide variety of hosts including mammals *in vivo* while birds, reptiles, and insects cell lines have also been infected in laboratory experiment [5, 6]. Whereas dog-mediated rabies is usually focused on by veterinary and public health organizations, rabies transmission by wildlife and as a reservoir on a landscape-scale has mostly been ignored [7]. It is critical knowledge that dogs play several roles in transmission of rabies. For example, domestic dogs can transmit the virus to other domestic animals and humans. Feral dogs and other feral animals that travel from human inhabitant areas to areas with wildlife in nearby jungles/forests can also transmit the virus to wildlife, and vice versa. Therefore, the cross-border movement of wild canids and their contact with domestic dogs pose a higher risk of rabies transmission, and its establishment in new localities, even in areas, which were originally rabies-free. Thus, even if a dog host is immunized, other animals of the surrounding landscape can transmit rabies and can maintain the viruses within different ecosystems and landscapes. We can find that those transmission details are widely overlooked and understudied.

**Figure 1:**
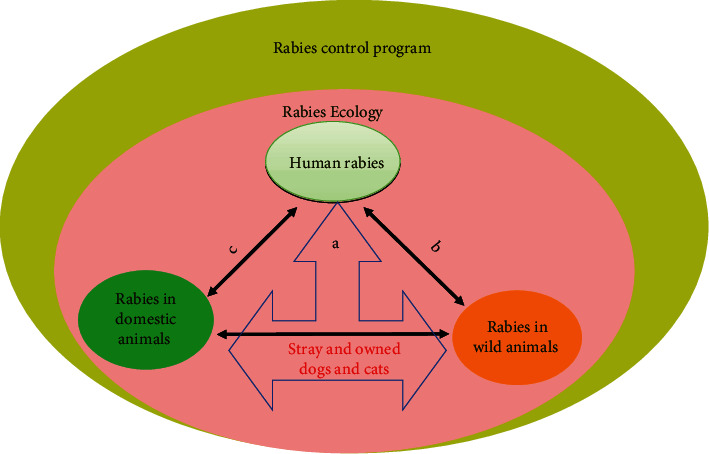
Sustainable rabies control plan. (a, b, c) Routes of rabies transmission.

Wild dogs, raccoons, foxes, jackals, mongoose, skunks, bats, ferret-badgers, and others have been shown to be the major reservoirs of rabies in wildlife [7–14]. Rabies has been reported to be maintained by European red foxes (*Vulpes vulpes*) and raccoon dogs (*Nyctereutes procyonoides*) in Europe; striped skunks (*Mephitis mephitis*), raccoons (*Procyon lotor*), red foxes, grey foxes (*Urocyon cinereoargenteus*), and coyotes (*Canis latrans*) in North America; side-striped and black-backed jackals (*Canis adustus*), mongoose species particularly the yellow mongooses (*Cynictis penicillata*) and bat-eared foxes (*Otocyon megalotis*)in southern Africa; and the Arctic foxes (*Vulpes lagopus*) in the northern polar areas [7, 14–16]. Even if much of the dog rabies has been eliminated from Canada and USA, rabies has been well maintained in wildlife species such as raccoons and arctic foxes [14, 17]. And for example in Oman as one example of many, sylvatic rabies is endemic, and fox (*Vulpes vulpes*) is the main reservoir and transmitter of rabies to domestic animals [18]. Mediouni et al. have reported the endemicity of rabies in Quebec, Canada, due to spillover of rabies from wildlife species to dogs [19]. In a surveillance study for a period of 10 years from 1994 to 2004 in South Carolina, foxes were reported to be the most common source for rabies exposure to people [20], and rabid skunk frequently interacting with dogs, cats, and livestock [20]. Rabies in red foxes and spill-over to domestic and wild animals in the United States was reported until mid-1990s [21]. Similarly, several studies have evidenced the spillover transmission of raccoon rabies to domestic animals [7, 14, 20–22]. These indicate the transmission and spillover of wildlife rabies to domestic animals including stray dogs, owned dogs, and cats (Figure 1). As a result of spillover, domestic animals may actually become potential vectors for raccoon rabies to humans as mentioned by Roseveare et al. [20]. However, no human casualties due to spillover of rabies virus directly from wild animals to humans have been reported [19]. There exists an ongoing risk of spillover of rabies from wild mammals to urban dogs (both stray and owned) population [23], livestock [24], and humans [25–28]. This spillover has also been observed in introduced foxes which act as carrier and spread disease to their prey animals such as lambs, goat kids, and others [24]. Interestingly, in Australia, the dingos and wild dogs co-occur with other species such as introduced feral cats, red foxes, and marsupials, all of which are potential threats for the spread of rabies. That is why canine rabies outbreak occurred in Australia along the Indonesian archipelago [29–33]. This can be explained by a research from Johnstone-Robertson et al., which have predicted the occurrence of 21% rabies in dingos (hybrids of domestic and wild canines) leading to its endemicity in Australia [34].

Another widely regulated wild species, roosting bats, also play a critical role in rabies transmission. For example, in the Americas, the majority of human rabies [35, 36] and livestock rabies are primarily due to the hematophagous bats such as vampire bats. In remote areas of the Amazon rainforest, the rise in incidence of human rabies is attributed to the common vampire bat (*Desmodus rotundus*) which sometimes feeds on humans [28]. In contrast, in Asia, Africa, and Oceania, bat-mediated human rabies cases are rare and it might be underreported because of the limited surveillance and characterization of viruses [37].

The risk of spillover from domestic animals to the wildlife cycle is evinced by molecular studies. For example, a rabies virus, isolated from the positive brain sample of mongoose, was similar to the virus originating from dogs or livestock, indicating a probable spillover transmission of canine rabies due to the mongoose interacting with an infected dog [38]. The spillover from domesticated animals to wildlife has been shown to pose a contributing risk for the conservation and protection of highly endangered carnivore species such as Ethiopian wolves (*Canis simensis*), African wild dogs (*Lycaon pictus*), and Blanford's fox (*Vulpes cana*) [37]. Rabies exposure in wildlife, domestic animals, and humans are associated with the economic impacts mainly related to vaccination costs and animal's death [39]. The direct cost of rabies is related to costs associated with postexposure prophylaxis (PEP), and livestock deaths are substantial, and they have been characterized in various studies [39–41]. Indirect costs are related to fear and subsequent disruption in economies due to rabies outbreaks. One study in 2007 to assess the cost-benefit analysis of oral rabies vaccine (ORV) to prevent and control skunk rabies in California, USA, has shown that the average cost of a single suspected rabies exposure was $4,000, and so this could squander millions of dollars each year [42]. The same research has shown that every dollar ($1) spent on wildlife rabies control and prevention, would in turn fetch benefits as high as $6.35 [42]. A similar cost-benefit analysis of raccoon rabies control programs in Quebec, Canada, reported a cumulative benefit-cost ratio ranging up to 1.55 [40], which indicates every dollar spent on the raccoon rabies control program saves up to $1.55 in preventable costs to the society [40]. Sterner et al. reviewed the modelling studies on economics of rabies vaccination in wildlife species such as red foxes, raccoons, skunks, and coyotes in Canada and USA. They showed that ORV programs in the wildlife sector could fetch a benefit-cost ratio of greater than 1 [41]. This indicates a significant economic benefit of rabies control in wildlife. Thus, it is worth spending resources in control of rabies in wildlife sector.

**Figure 2:**
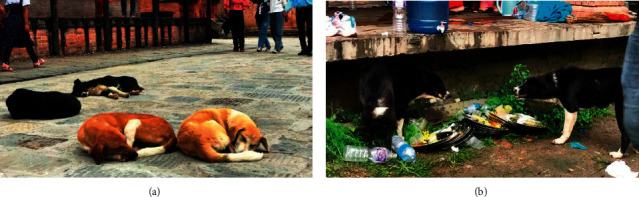
(a, b) Close interface between dogs and humans (photos kindly provided by Dikpal Karmacharya, Third Pole Conservancy, Bhaktapur, Nepal). The photographs represent the common situation of human-domestic animal existence globally.

The threat of zoonotic diseases spilling-over into the wild and domestic animal-human interface is increasing. That is due to decreased natural habitat and increased interaction between animals and humans in and around national parks, conservation areas, and game parks [43] (Figures 2(a) and 2(b)). Some countries in Asia and Africa are at an increased risk of incursion of rabies from wildlife due to abundant domestic and wild dog populations as well as the close interface among vectors and host [44–46]. Particularly, indigenous people who live in close contact to wildlife reserves, sanctuaries, and national parks are reported to have been at higher risk of acquiring rabies from wild animals [45, 46]. Also, religious people, visitors, and tourists to temple areas of the world, for example, those in the Kathmandu valley in Nepal or Lumbini, Nepal, are at high risk of acquiring rabies from monkeys via bites and scratches, although there are no data collected on the transmission of rabies from monkeys to humans so far [47]. Due to the close interface between wild animals and humans in rural settlements and villages, attacks and the subsequent transmission of rabies from infected wildlife have been reported [25–27]. Different countries have enforced various programs to control rabies in domestic animals, but there is a recognized need for rabies control and management originating from wild animals [48–51]. It is evident that the existing rabies control projects are mainly focused on vaccinating city dwelling cats and dogs and its human populations, whereas the burden and transmission of rabies sourced from wild animals is higher in rural and wilderness areas [28, 52–54]. However, it has received little or no attention and often goes unnoticed. The study by the University of Alaska group shows urbanized and industrial areas as hotspots [14]. The overall burden of rabies in wildlife is underestimated [46] and rabies control programs are almost nonexistent in low and middle-income nations such as Nepal, India, and other South Asian countries [37, 55]. The rabies-endemic underdeveloped countries usually have not addressed the role of wildlife in rabies epidemiology or they lack clarity regarding the control of rabies in wild animals, despite having clear guidelines set up by GARC [56, 57] and WHO 2018. Those are often not followed. This could be attributed to uncertainty regarding disease dynamics, which complicates to identify the best management approaches [43, 58], as well as difficulty in advocating and implementing control of rabies in wild animals and such reservoirs. Another important reason is the limited resource availability such as budget, expert manpower, which is prioritized for urban rabies control programs compared to rabies control in wildlife and wild landscapes. The use of oral baits, as a means of administering rabies vaccine, has been quite successful to control rabies in red foxes in Europe [59], with Arctic foxes in Canada [60, 61] and for raccoons in USA [61, 62]. While the control programs in wild canines have been somewhat successful, the actual elimination of bat rabies is not really feasible at the moment due to lack of effective vaccines and delivery system for bat vaccination [37]. Complex bat biology, e.g., roosting in trees and migratory long distance, contributes to such difficulties in rabies control. Interestingly, research is ongoing to vaccinate bats with recombinant rabies vaccine with the Australian bat lyssavirus glycoprotein [62, 63]. If successful, it could be a potential candidate to control rabies from bats, and subsequent transmission to feral foxes and other wild carnivores.

It is important to emphasize that ‘The Global Strategic Agreement and Plan to End Human Deaths from Dog-mediated Rabies by 2030' (Zero by 30) and eliminate rabies by the WHO, the FAO, the OIE, and GARC is critical [3]. However, we suspect that without considering the rabies in wildlife, the above plan would not really be feasible, hardly realistic. While claiming zero human death by rabies by 2030 might be ideal, it is not possible indeed. The underlying causes for this problem mainly include already the central role of dogs. First, domestic dogs and humans have a shared habitat, and so have the feral dogs and wildlife faunae. Second, it is not easy to separate and divorce dogs and wilderness from each other in the ecology and zoonotic diseases in landscapes and the Anthropocene. Therefore, regarding the Rabies Goal, it is not wise for any organization or country to do pie in the sky. Furthermore, some people refer to it as a hoax especially due to the failure paths of various earlier but boldly promoted goals, e. g., Biodiversity Goals by 2010 [64], Paris Climate Agreement Goals [65, 66], and Sustainable Development Goals by 2030 [67]. Therefore, unless one sticks to the words, and comes up with trusted and reality aims for the public, the Zero by 30 Policy is just paperwork, and nobody actually cares about it harming its own goals and support.

## 3. Conclusions

Controlling rabies still needs a more holistic approach, and an ecologic view is essential to be serious. The study of reservoirs, big data mining, open access predictions, and subsequent outbreaks of any zoonotic disease must be addressed as mentioned, for instance by Gulyaeva et al. [68]. Here, we conclude that the control of rabies at the domestic-wild animal interface on a landscape-scale is vital, and the inclusion of wild animals in rabies control programs is crucial. Otherwise, the control of rabies in domestic animals and humans will not achieve tangible results and cannot be effective. Epidemiological investigations determining the role and significance of wildlife on a landscape-scale for rabies is essential, and this should be a priority of concerned governments and nongovernmental organizations. We propose that an integrated surveillance program with open access data be implemented at the human-domestic animal-wild animal interface so that an effective early detection and identification of susceptible animal populations, and control and prevention of rabies around the world will be possible.

## Data Availability

Data sharing is not applicable to this article as no new data were created or analyzed in this study.
